# A survey of cancer patients’ unmet information and coordination needs in handovers – a cross-sectional study

**DOI:** 10.1186/1756-0500-6-378

**Published:** 2013-09-25

**Authors:** Eva Gad Søndergaard, Bettina Haastrup Grøne, Christian Nielsen Wulff, Pia Veldt Larsen, Jens Søndergaard

**Affiliations:** 1 Unit for Health Promotion Research in Esbjerg, University of Southern Denmark, Niels Bohrs Vej 9-10, 6700 Esbjerg, Denmark; 2 Research Unit for General Practice, Aarhus University, Bartholins Allé 2, 8000 Aarhus C, Denmark; 3 Research Unit of General Practice in Odense, University of Southern Denmark, J.B Winsløws Vej 9A, 5000 Odense C, Denmark

**Keywords:** Cancer, Need, Information, Coordination, Handovers

## Abstract

**Background:**

The care responsibilities for cancer patients are frequently handed over from one healthcare professional to another. These handovers are known to pose a threat to the safety of patients and the efficiency of the healthcare system. Little is known about specific needs of cancer patients in handovers. The objectives of this study were to examine cancer patients’ unmet needs for information and coordination in handovers and to analyse the association between patients’ demographic and clinical characteristics and unmet information and coordination needs.

**Methods:**

Cancer patients treated in an oncology and a surgery outpatient setting completed a questionnaire developed to examine unmet information and coordination needs of cancer patients in handovers. Associations between unmet needs and comorbidity, treatment type, time since diagnosis, gender, age, and education in various handover situations were analysed.

**Results:**

Of 250 eligible patients 131 participated (response rate of 52%). Overall, 18% of patients had unmet coordination needs and 18% had unmet information needs.

Hospital discharge was the type of handover where patients most frequently reported unmet information needs (18%). Unmet coordination needs were most frequently reported in handovers between different hospitals (19%) and in handovers between hospital and general practice (18%). In general, age and education were statistically significantly associated with reporting unmet needs, where patients younger than 60 years and patients with a higher education were more likely to express unmet needs.

**Conclusions:**

The findings indicate room for improvements regarding exchange of information and coordination between healthcare professionals, and between healthcare professionals and patients.

## Background

A high level of specialisation and fragmentation characterise today’s healthcare systems [1]. The care responsibility for the patient is frequently handed over between healthcare professionals, and during this process vital information may be lost, resulting in patient-experienced fragmentations of care, mistakes, oversights [2], and systematic errors, prompting wasted time and resources [3]. Consequently, patients might feel left in limbo, which can represent a barrier to the feeling of safety and wellbeing [4-6]. A handover is defined as “A situation in which the responsibility for a patient’s diagnosis, treatment, and care is transferred fully or partly, temporarily or permanently from one healthcare professional to another” [3]. Inadvertent events occurring during handovers may be related to lack of information, miscommunication, unclear care responsibility, or inadequate organisational framework [3,7,8]. Handovers may thus pose a threat to the safety of patients and the efficiency of healthcare systems.

Cancer is a complex disease including numerous handovers during an often protracted cancer course, which can be a challenge to any healthcare system [1,9]. Little is known about the patients’ perspective during handovers [10]. Thus, it is relevant to examine whether the needs of cancer patients in handovers are met in order to ensure that they experience an optimal cancer care trajectory. Apparently, cancer patients commonly have unmet needs within the domains of information and coordination [11-13]. Information needs are to a great extent related to disease and specific treatment information, such as physical changes, side-effects, metastasis, and recovery [14,15].

Coordination needs are often related to continuity of care, that is, care delivered by different healthcare professionals in a coherent, logical, and timely fashion, consistent with the patients’ needs [16]. Insufficient coordination is often comprised of insufficient information, communication, and cooperation between healthcare professionals [16-18]. Studies show that patients do not know where to enquire information, and they experience a lack of systematic rehabilitation offers and insufficient cooperation between hospitals [18].

Patients’ needs vary according to demographic and clinical patient characteristics [13,19-21]. Furthermore, studies have indicated that the kind of treatment received and the presence of comorbidity influence patient rehabilitation needs [20]. Also, patient needs depend on the phase in the cancer care trajectory [11,21,22].

Studies of handovers are predominantly concerned with best practice at an organisational and structural level and only to a lesser extent include the patients’ perspective [10]. In-depth knowledge in the field of cancer patients’ experiences of needs in handovers may contribute to quality improvements in the cancer care trajectory. To our knowledge, no validated questionnaire has been developed to evaluate the information and coordination needs of cancer patients specific for handovers. The purpose of this study was to 1) examine the unmet information and coordination needs experienced by cancer patients in handovers during their cancer care trajectories, and 2) analyse the associations between demographic and clinical patient characteristics and unmet information and coordination needs.

## Material and methods

This study employed a cross-sectional survey design using a questionnaire developed to examine cancer patients’ unmet information and coordination needs in handovers. The survey was conducted in a sample of patients with breast, colorectal, and prostate cancer recruited from the oncology and the surgery outpatient clinics at Hospital Southwest Jutland in Esbjerg, which is a provincial hospital in the Region of Southern Denmark.

### Study population

The study population comprised all patients attending the two outpatient clinics described above in the period 2 January – 1 March 2012. Patients were eligible if they were 18 years or older and if they were more than three months post-diagnosis. Patients were excluded if a research nurse (RN) assessed that patients were unable to understand and complete a Danish questionnaire or were judged to be cognitive impaired. Eligible patients who came for a consultation were informed about the study by an RN and given the questionnaire. Participants returned the completed questionnaire in a prepaid return envelope together with a signed letter of consent. Information on sex, age, years since diagnosis, cancer type and treatment were retrieved by RNs from medical records.

### Questionnaire

The self-administered questionnaire comprised 50 items within the domains of information and coordination and seven items related to sociodemographic characteristics. The items on information and coordination focused on various handover situations (handovers between primary and secondary sector, between internal hospital departments and between hospitals). Furthermore, the questionnaire included items of clinical relevance to outpatient clinics, and patients could add supplementary comments.

Respondents indicated their level of needs on a four-point Likert scale with the following response categories: “Strongly agree”, “Agree”, “Disagree”, and “Strongly disagree”. The responses were coded with the values one (“Strongly agree”) to four (“Strongly disagree”). Furthermore, a “Don’t know/Not relevant” response option was given. In order to handle missing values, a patient score was calculated as the mean of the responses corresponding to the eight scales of needs. In the analyses each patient’s score was dichotomised, using a score of two as cut-point. A mean below or equal to two was equivalent to “Strongly agree” or “Agree”. Likewise, a mean above two was equivalent to “Disagree” or “Strongly disagree”. A score less than two was categorised as having “no unmet needs” and a value equal to or greater than two was categorised as “having unmet needs”.

The items regarding information formed three handover specific subscales; hospital discharge (eight items), handovers between internal hospital departments (ten items), and handovers between hospitals (nine items). The 27 item together formed an overall information scale.

The items regarding coordination formed four handover specific subscales; between internal hospital departments (six items), between hospitals (seven items), and between hospital and general practitioner (GP) (seven items). The 20 items together formed an overall coordination scale. Table 1 lists a sample of the items.

**Table 1:** Selected items within the domains of information and coordination translated from Danish

**So far, I have received sufficient information regarding:**	**I consider my general practitioner to be sufficiently informed about:**
…my cancer disease and treatment	…my examinations and treatments
…transport and accommodation	…check-ups
…waiting time	…test results
…possibilities for social assistance	…complications
…possibilities for counseling regarding my job situation	…my physical rehabilitation plan
	…my psychological condition
…possible changes regarding carrying out daily activities e.g. cleaning, grocery shopping, and personal hygiene	
…possibilities for counseling outside the hospital e.g. The Danish Cancer Society	
…impact of lifestyle on my cancer disease e.g. diet, physical activity, smoking and alcohol consumption	
…possible psychological reactions	
…coping with my disease and my next of kin e.g. family and friends	
…my sexuality	
…alternative treatment e.g. reflexology, acupuncture, healing, and homeopathy	

### Development and piloting of patient questionnaire

The questionnaire was developed through different approaches. Classification of needs in the two domains was made based on a comprehensive literature search of needs of cancer patients. Relevant items from various existing instruments for measuring needs of cancer patients [6,13,18,23-27], were adjusted to apply in the context of this study. Researchers in the field of cancer care and external reviewers assessed the contents, clarity of wording, and comprehensiveness of the items [28]. Three pilot tests, including a total of 25 cancer patients, were conducted. Piloting included time used to fill in the questionnaire and interviews with the patients about comprehension of the items [29]. Based on the three pilot tests the questionnaire was revised. A final pilot test, including hand-outs of the questionnaire to 63 cancer patients, tested the hand-out procedure and the willingness to participate (response rate of 67%). Patients from the pilot tests were not included in the final study sample.

### Statistical analysis

Descriptive statistics were calculated, comparing participants with non-participants. This study considers a total of eight different outcome variables concerning needs in handovers. Four outcomes relate to information needs (three subscales as well as overall information need), and four outcomes relate to coordination needs (three subscales as well as overall coordination need). Univariate logistic regression was used to analyse the crude associations between each outcome and each of the independent variables comorbidity, treatment type, time since diagnosis, gender, age, and education, respectively. Education was dichotomised as lower education (less than three years of continuing education), and higher education (three years or more of continuing education).

Due to the limited number of participants it was only possible to adjust for a small number of potential confounders. It was decided to adjust for age, gender and education as the literature search identified these as important potential confounders. Cancer type was not included as a confounder, since some of the cancer-diagnoses in the study are gender-specific. In order to adjust for the three potential confounders, we conducted separate multiple logistic regressions analysing the associations between each of the outcomes and each of the independent variables, adjusting for age, gender and education. Gender and age are known to be associated with patient needs, as women and younger patients are generally more likely to report unmet needs [13,19-21]. The patients’ levels of education has shown to influence rehabilitation and information needs [19-21].

A p-value below 0.05 was considered statistically significant. Internal reliability was assessed using Cronbach’s α and Pearson correlation. EpiData (The EpiData Association, Odense, Denmark) was used for entering data and all statistical analyses were performed using Stata 12 (StataCorp LP, College Station, Texas, USA).

### Ethics

The study was approved by the Danish Data Protection Agency and the senior consultants at the Oncology Department and at the Surgery Department at Hospital Southwest Jutland, Esbjerg. According to the Danish Research Ethics Committee System we did not need the committee’s approval [30,31].

## Results

**Figure 1:**
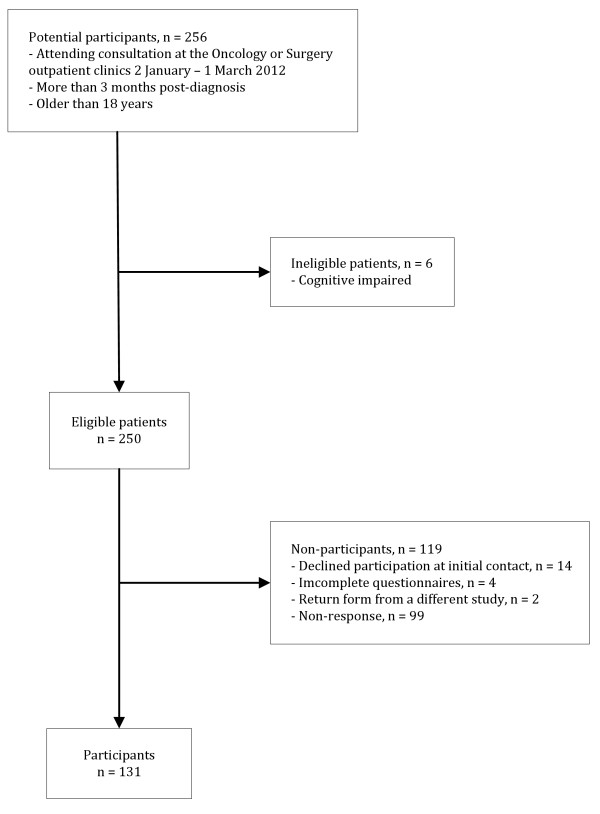
Study flowchart.

Figure 1 illustrates the patient flow. The RNs identified 256 potential participants. Of these, 6 were excluded in accordance with the exclusion criteria. A total of 131 completed questionnaires were returned, yielding a response rate of 52%.

The internal reliability of each of the subscales was high as Cronbach’s alphas were above 0.93. Table 2 depicts Cronbach’s alphas for all eight scales.

**Table 2:** Overview of Cronbach’s alpha values

**Scale**	**Cronbach’s alpha value**
Overall unmet information needs	0,97
Unmet information needs at hospital discharge	0,93
Unmet information needs in handovers between internal hospital departments	0,90
Unmet informations needs in handovers between hospitals	0,93
Overall unmet coordination needs	0,97
Unmet coordination needs in handovers between hospital and GP	0,97
Unmet coordination needs in handovers between internal hospital departments	0,93
Unmet coordination needs in handovers between hospitals	0,96

### Patient characteristics

Table 3 presents the sociodemographic and clinical characteristics of participants and non-participants. There were only minor differences between the two groups. The proportion of patients diagnosed more than three years previously was a little lower among participants (54%) than non-participants (61%). Further, participants were slightly more likely to have had hormone treatment (77% vs. 72%).

**Table 3:** Demographic and clinical characteristics of participants and non-participants

	**Participants, *n (%)***	**Non-participants, *n (%)***
*All*	131 (52.4)	119 (47.6)
*Demographic characteristics*		
Gender*		
Female	98 (75)	86 (72)
Male	33 (25)	33 (28)
Age*	*65 (9.5), 41-86*	*66 (11.4), 39-90*
*Mean (SD), min-max*		
<60 years	38 (29)	40 (34)
≥60 years	93 (71)	78 (66)
Years since diagnosis*	*3.09 (1.65-6.01)*	*3.89 (2.03-6.76)*
*Median (IQR)*		
<3 years	60 (46)	46 (39)
≥3 years	70 (54)	71 (61)
*Marital status*^*#*^		
Not living with a partner	28 (22)	-
Living with a partner	102 (78)	-
*Children*^*#*^		
No children living at home	114 (89)	-
Children living at home	14 (11)	-
*Education*^*#*^		
No higher education	70 (57)	-
Higher education	56 (44)	-
*Occupation*^*#*^		
Not retired	47 (37)	-
Retired	80 (63)	-
*Clinical characteristics*		
*Comorbidity*		
No comorbidity	40 (32)	-
Comorbidity	95 (68)	-
Cancer type*		
Breast	90 (71)	82 (69)
Prostate	27 (21)	22 (19)
Colorectal	10 (8)	11 (9)
Other	0 (0)	3 (3)
Treatment*		
Surgery	96 (74)	92 (78)
Radiation therapy	79 (61)	66 (56)
Chemotherapy	68 (52)	64 (54)
Hormone therapy	100 (77)	85 (72)
Other pharmaceutical	17 (13)	13 (11)
Other treatments	3 (2)	2 (2)
No treatment	4 (3)	2 (2)

*Information on sex, age, years since diagnosis, cancer type and treatment were retrieved by RNs from medical records at the time of assessment.

^#^Information available only on included patients because information was self-reported, i.e. from the patient questionnaire.

### Prevalences and associations

A total of 23 (18%) patients reported unmet information needs, while 24 (18%) reported unmet coordination needs. Unmet information and coordination needs were statistically significantly associated with age and education. Patients younger than 60 years of age and patients with a higher education had higher odds of indicating unmet needs than patients older than 60 years and patients without a higher education, respectively (cf. Tables 4 and 5).

**Table 4:** Unmet information needs

	**Overall unmet information needs**			**Unmet information needs at hospital discharge**^**#**^			**Unmet information needs in handovers between internal hospital departments**^**##**^			**Unmet information needs in handovers between hospitals**^**###**^		
	**N (%)**	**OR (95% CI)**	**OR**_**adj**_^**a **^**(95% CI)**	**N (%)**	**OR (95% CI)**	**OR**_**adj**_^**a **^**(95% CI)**	**N (%)**	**OR (95% CI)**	**OR**_**adj**_^**a **^**(95% CI)**	**N (%)**	**OR (95% CI)**	**OR**_**adj**_^**a **^**(95% CI)**
*Demographic characteristics*												
Gender												
Women	19 (19%)	1.00	1.00	14 (16%)	1.00	1.00	8 (12%)	1.00	1.00	12 (13%)	1.00	1.00
Men	4 (12%)	0.57 (0.18; 1.83)	1.10 (0.31; 3.93)	4 (25%)	1.71 (0.48; 6.09)	2.24 (0.58; 8.64)	3 (21%)	2.05 (0.47; 8.94)	3.31 (0.66; 16.59)	4 (12%)	0.97 (0.29; 3.23)	2.77 (0.63; 11.20)
Age												
<60 years of age	13 (33%)	1.00	1.00	9 (26%)	1.00	1.00	6 (23%)	1.00	1.00	9 (24%)	1.00	1.00
≥60 years of age	10 (11%)	0.24*(0.10; 0.62)	0.26*(0.10; 0.70)	9 (13%)	0.42 (0.15; 1.19)	0.36 (0.12; 1.07)	5 (9%)	0.33 (0.09; 1.19)	0.26 (0.07; 1.05)	7 (8%)	0.27*(0.09; 0.79)	0.23*(0.07; 0.80)
Marital status												
No partner	8 (29%)	1.00	1.00	7 (29%)	1.00	1.00	2 (13%)	1.00	1.00	5 (19%)	1.00	1.00
Partner	15 (15%)	0.43 (0.16; 1.16)	0.39 (0.13; 1.16)	11 (14%)	0.40 (0.13; 1.18)	0.39 (0.12; 1.24)	9 (13%)	1.01 (0.19; 5.23)	0.63 (0.10; 3.78)	11 (11%)	0.54 (0.17; 1.71)	0.47 (0.12; 1.88)
Education												
Not higher education	7 (10%)	1.00	1.00	6 (12%)	1.00	1.00	4 (9%)	1.00	1.00	2 (3%)	1.00	1.00
Higher education	16 (29%)	3.60*(1.36; 9.52)	3.41*(1.23; 9.44)	12 (24%)	2.43 (0.83; 7.11)	2.53 (0.84; 7.59)	7 (19%)	2.28 (0.61; 8.49)	2.72 (0.67; 11.09)	14 (26%)	11.90*(2.57; 55.08)	13.88*(2.78; 67.23)
*Clinical characteristics*												
Comorbidity												
No comorbidity	8 (20%)	1.00	1.00	6 (19%)	1.00	1.00	4 (14%)	1. 00	1.00	4 (10%)	1.00	1.00
Yes comorbidity	15 (16%)	0.79 (0.30; 2.07)	1.35 (0.45; 4.05)	12 (18%)	0.93 (0.31; 2.75)	1.33 (0.40; 4.38)	7 (14%)	1.02 (0.27; 3.82)	2.17 (0.43; 10.91)	12 (14%)	1.46 (0.44; 4.85)	3.51 (0.82; 15.09)
*Treatment*												
Surgery												
No surgery	3 (9%)	1.00	1.00	3 (21%)	1.00	1.00	3 (20%)	0.55	1.00	3 (9%)	1.00	1.00
Surgery	20 (21%)	2.72 (0.75; 9.81)	2.98 (0.35; 25.77)	15 (17%)	0.76 (0.19; 3.07)	1.21 (0.12; 12.24)	8 (12%)	0.54 (0.13; 2.35)	0.63 (0.02; 15.89)	13 (14%)	1.66 (0.44; 6.22)	3.30 (0.23; 46.73)
Radiation												
No radiation	10 (20%)	1.00	1.00	10 (30%)	1.00	1.00	2 (15%)	1.00	1.00	7 (14%)	1.00	1.00
Radiation	13 (16%)	0.81 (0.32; 2.01)	0.73 (0.26; 2.01)	8 (12%)	0.31*(0.11; 0.87)	0.33 (0.11; 1.01)	9 (13%)	0.83 (0.16; 4.35)	0.75 (0.13; 4.42)	9 (12%)	0.83 (0.29; 2.40)	0.93 (0.28; 3.11)
Chemotherapy												
No chemotherapy	8 (13%)	1.00	1.00	7 (17%)	1.00	1.00	5 (14%)	1.00	1.00	8 (13%)	1.00	1.00
Chemotherapy	15 (22%)	1.91 (0.75; 4.88)	1.10 (0.30; 4.06)	11 (18%)	1.09 (0.38; 3.09)	1.21 (0.31; 4.82)	6 (13%)	0.98 (0.27; 3.53)	1.15 (0.18; 7.53)	8 (12%)	0.93 (0.33; 2.65)	0.43 (0.08; 2.26)
Hormones												
No hormones	8 (27%)	1.00	1.00	6 (26%)	1.00	1.00	3 (19%)	1.00	1.00	3 (10%)	1.00	1.00
Hormones	15 (15%)	0.49 (0.18; 1.29)	0.45 (0.15; 1.33)	12 (15%)	0.52 (0.17; 1.57)	0.50 (0.15; 1.67)	8 (12%)	0.60 (0.14; 2.57)	0.50 (0.10; 2.52)	13 (13%)	1.31 (0.35; 4.95)	1.38 (0.30; 6.40)
*Years since diagnosis*												
<3 years	11 (18%)	1.00	1.00	7 (16%)	1.00	1.00	6 (15%)	1.00	1.00	9 (16%)	1.00	1.00
≥3 years	12 (17%)	0.92 (0.37; 2.27)	0.99(0.35; 2.78)	11 (19%)	1.26 (0.45;3.58)	1.65(0.50; 5.47)	5 (12%)	0.81 (0.23; 2.90)	1.00 (0.24; 4.15)	7 (10%)	0.60 (0.21; 1.74)	0.81(0.23; 2.87)

^a^Adjusted for age, gender and education, * P < 0.050, ^#^Consists of 102 patient responses, ^##^Consists of 82 patient responses, ^###^Consists 129 patient responses.

**Table 5:** Unmet coordination needs

	**Overall unmet coordination needs**			**Unmet coordination needs in handovers between hospital and GP**^**#**^			**Unmet coordination needs in handovers between internal hospital departments**^**##**^			**Unmet coordination needs at handovers between hospitals**^**###**^		
	**N (%)**	**OR (95% CI)**	**OR**_**adj**_^**a **^**(95% CI)**	**N (%)**	**OR (95% CI)**	**OR**_**adj**_^**a **^**(95% CI)**	**N (%)**	**OR (95% CI)**	**OR**_**adj**_^**a **^**(95% CI)**	**N (%)**	**OR (95% CI)**	**OR**_**adj**_^**a **^**(95% CI)**
*Demographic characteristics*												
Gender												
Women	19 (19%)	1.00	1.00	17 (20%)	1.00	1.00	8 (9%)	1.00	1.00	14 (18%)	1.00	1.00
Men	5 (15%)	0.74 (0.25; 2.18)	1.10 (0.31; 3.93)	4 (13%)	0.63 (0.19; 2.06)	0.88 (0.25; 3.09)	2 (6%)	0.74 (0.15; 3.69)	2.53 (0.37; 17.47)	5 (24%)	1.45 (0.46; 4.62)	1.85^a^(0.48; 7.07)
Age												
<60 years of age	14 (36%)	1.00	1.00	9 (29%)	1.00	1.00	7 (19%)	1.00	1.00	9 (29%)	1.00	1.00
≥60 years of age	10 (11%)	0.22*(0.09; 0.55)	0.26*(0.10; 0.70)	12 (14%)	0.40 (0.15; 1.06)	0.44 (0.16; 1.25)	3 (3%)	0.16*(0.04; 0.65)	0.14*(0.03; 0.68)	10 (14%)	0.41 (0.15; 1.15)	0.46^b^(0.15; 1.37)
Marital status												
No Partner	5 (18%)	1.00	1.00	3 (12%)	1.00	1.00	2 (7%)	1.00	1.00	4 (18%)	1.00	1.00
Partner	19 (19%)	1.05 (0.33; 3.13)	1.06 (0.32; 3.50)	18 (20%)	1.81 (0.49; 6.71)	2.05 (0.51; 8.16)	8 (8%)	1.12 (0.22; 5.59)	1.52 (0.24; 9.55)	15 (19%)	1.07 (0.32; 3.63)	0.91^c^(0.25; 3.28)
Education												
Not higher education	7 (10%)	1.00	1.00	8 (12%)	1.00	1.00	1 (1%)	1.00	1.00	7 (13%)	1.00	1.00
Higher education	16 (29%)	3.06*(1.36; 9.52)	3.41*(1.23; 9.44)	13 (28%)	2.91*(1.09; 7.73)	2.82*(1.04; 7.63)	9 (17%)	13.02*(1.60; 106.25)	14.22*(1.61; 125.79)	11 (26%)	2.31 (0.81; 6.59)	2.47^d^(0.82; 7.37)
*Clinical characteristics*												
Comorbidity												
No comorbidity	10 (25%)	1.00	1.00	6 (18%)	1.00	1.00	5 (13%)	1. 00	1.00	7 (21%)	1.00	1.00
Yes comorbidity	12 (14%)	0.49 (0.19; 1.26)	0.75 (0.26; 2.13)	14 (18%)	0.99 (0.35; 2.84)	1.24 (0.41; 3.80)	5 (6%)	0.44 (0.12; 1.63)	0.83 (0.19; 3.67)	11 (17%)	0.82 (0.28; 2.34)	1.15^c^(0.35; 3.78)
*Treatment*												
Surgery												
No surgery	3 (9%)	1.00	1.00	5 (16%)	1.00	1.00	2 (6%)	0.55	1.00	3 (14%)	1.00	1.00
Surgery	21 (22%)	2.89 (0.80; 10.41)	2.98 (0.35; 25.77)	16 (19%)	1.27 (0.42; 3.81)	0.37 (0.05; 3.04)	8 (9%)	1.43 (0.29; 7.08)	0.63 (0.04; 9.04)	16 (21%)	1.55 (0.41; 5.91)	6.06^c^(0.33; 110.58)
Radiation												
No radiation	9 (18%)	1.00	1.00	6 (13%)	1.00	1.00	4 (8%)	1.00	1.00	7 (27%)	1.00	1.00
Radiation	15 (19%)	1.09 (0.44; 2.73)	1.26 (0.44; 3.56)	15 (21%)	1.74 (0.62; 4.88)	1.80 (0.60; 5.42)	6 (8%)	0.96 (0.26; 3.58)	1.14 (0.26; 5.01)	12 (16%)	0.53 (0.18; 1.55)	0.57^c^(0.18; 1.82)
Chemotherapy												
No chemotherapy	8 (13%)	1.00	1.00	7 (12%)	1.00	1.00	4 (7%)	1.00	1.00	10 (23%)	1.00	1.00
Chemotherapy	16 (24%)	2.08 (0.82; 5.27)	1.10 (0.30; 4.06)	14 (24%)	2.22 (0.82; 6.00)	1.91 (0.49; 7.44)	6 (9%)	1.35 (0.36; 5.04)	0.54 (0.07; 4.17)	9 (16%)	0.67 (0.24; 1.82)	0.34^c^(0.08; 1.38)
Hormones												
No hormones	9 (30%)	1.00	1.00	9 (38%)	1.00	1.00	2 (7%)	1.00	1.00	5 (23%)	1.00	1.00
Hormones	15 (15%)	0.41 (0.16; 1.07)	0.45 (0.15; 1.33)	12 (13%)	0.25*(0.09; 0.70)	0.21*(0.07; 0.66)	8 (8%)	1.24 (0.25; 6.20)	1.39 (0.23; 8.55)	14 (18%)	0.75 (0.24; 2.39)	0.87^c^(0.24; 3.11)
*Years since diagnosis*												
<3 years	12 (20%)	1.00	1.00	11 (22%)	1.00	1.00	6 (10%)	1.00	1.00	8 (17%)	1.00	1.00
≥3 years	12 (17%)	0.83 (0.34; 2.01)	0.99 (0.35; 2.78)	10 (15%)	0.66 (0.26; 1.71)	0.61 (0.21; 1.76)	4 (6%)	0.56 (0.15; 2.10)	0.73 (0.16; 3.38)	11 (22%)	1.38 (0.50; 3.38)	1.98^c^(0.63; 6.25)

^a^Adjusted for age, gender and education, *P < 0.050, ^#^Consists of 117 patient responses, ^##^Consists of 125 patient responses, ^###^Consists of 100 patient responses.

Hospital discharge was the handover that generated the highest level of unmet information needs (18%) compared to handovers between internal hospital departments (13%) and between hospitals (12%) (cf. Table 4). The highest level of unmet coordination needs was reported in handovers between hospitals (19%) and between hospital and GP (18%). Only few patients (8%) indicated unmet coordination needs in handovers between hospital departments (cf. Table 5).

Patients who underwent surgery or received chemotherapy had higher odds of reporting information and coordination needs than patients who did not undergo surgery and did not receive chemotherapy.

No general tendencies were observed in relation to gender, comorbidity, time since diagnosis, radiation therapy, and hormone treatment.

## Discussion

### Main findings

Approximately every sixth patient reported unmet coordination needs and 18% reported unmet information needs. Hospital discharge was the handover with the highest level of unmet information needs. Both in handovers between hospital and GP and handovers between hospitals unmet coordination needs were relatively frequent. Age and education were statistically significantly associated with reporting unmet needs in both of the overall scales and in several of the subscales. Thus, patients younger than 60 years and patients with a higher education were more likely to express unmet needs than patients older than 60 years and patients without a higher education, respectively.

### Strengths and weaknesses

A strength of the study is that the final questionnaire was developed based on previously used instruments for measuring needs of cancer patients and it had been thoroughly pilot tested. Furthermore, Cronbach’s α showed high internal reliability of the scales for each domain. However, some of the Cronbach’s αs were very high (α > 0.95) suggesting that there might be redundant items in these scales. Cronbach’s αs do not provide a thorough assessment of validity or general reliability thus a lack of rigorous psychometric evaluation of the survey can be considered a weakness.

Registering eligible patients and introducing them to the survey plus collecting journal information meant extra work for the already busy RNs. Thus an outpatient setting might not be optimal for a survey of this type. In order not to let the surroundings (i.e. the busy outpatient clinic) influence responses patients were recommended by the RNs to fill in the questionnaire at home. This however did not allow for sending reminders to potential participants which might have improved the response rate.

It is reasonable to interpret the indication of disagreement as an unmet need as the questionnaire use the wording “sufficiently”. However the questions used in the study do not directly address whether the patients actually wanted help with aspects of information and coordination.

A comparison of participants and non-participants revealed only minor differences. Breast cancer was the most prevalent diagnosis both among participants (71%) and non-participants (69%) exceeding the national breast cancer prevalence of 23% among Danish cancer patients [32]. This is partly explained by the specialisation of Danish hospital departments. Thus, treatment and follow-up of patients suffering from other cancer types in the area of Esbjerg often take place at other hospitals. Among participants patients three years post diagnosis were underrepresented compared to non-participants. This might be due to patients not wishing to revisit earlier phases of their cancer care trajectory or believing that their experiences were outdated and thus not relevant for the study.

Our study included a combination of questionnaire data and data obtained from medical records. It would have been interesting to also include data on e.g. depression, because this characteristic may be associated with unmet needs [33].

The statistical analyses adjusted for some important confounders. However, for some of the analyses the number of participants was limited resulting in reduced statistical precision reflected in the wide CIs.

As this study included patients in different phases of their cancer care trajectory, and as there are variations in the experiences of cancer patients, the recollection of experienced needs may vary. Generalisation of the results to other patients groups should be made with caution, because research has shown that patterns of needs depend on the phases in the care trajectory [11,21,22]. However, it is reasonable to assume that the tendencies indicated by the present study can be guiding for unmet information and coordination needs in similar contexts.

### Comparison with other studies

Our study found that the highest level of unmet information needs was reported in relation to hospital discharge. This is in agreement with a Danish study which indicated that patients discharged experienced a lack of information and that the information given at the discharge was inconsistent between patients. Additionally, patients requested personalised information on possible side effects and wished to be treated by only a few different healthcare providers [34].

Knowledge of what patients find important or beneficial in care coordination is sparse [35]. Studies relating to coordination of care are often focused on cooperation which is concerned with the structural organisation, or on patient preferences for care and involvement in the decision-making process. Despite increased focus on care coordination, a recent Danish study of outpatients’ experiences (n = 96,860) indicated that there is still substantial room for improvement in relation to handovers [36]. The study found that 15% of patients experienced that their GP was not well-informed about the treatment of the patient. Approximately 7% experienced poor coordination between hospital departments and between hospital and municipality. Although these findings are not cancer-specific and are concerned with cooperation, rather than coordination and information, they support our results. Likewise, Grønvold et al. (2006) conducted a comprehensive Danish study (n = 1,518) of the needs and experiences of cancer patients. They found that a large proportion of cancer patients did not experience optimal cooperation between hospitals. Some 20% of the patients did not experience satisfactory cooperation between hospitals and 20% did not experience optimal cooperation between hospital and GP. Furthermore, patients did not find information to be timely, and frequently requested additional information. In accordance with our study, patients younger than 60 years old and patients with a higher education reported a higher level of unmet needs [18]. The age variation in needs might be explained by today’s younger patients being more explicit about their specific needs [19]. Furthermore, younger patients are more critical towards authorities, demanding dialogue, respect and good service [37]. As cancer prevalence is higher among the elderly, younger patients are less likely to have age-related peers going through a similar situation. This in itself could be isolating and reduces the opportunity of younger patients to gain insight and information from peers in a similar situation. Thus, it is possible that younger patients have different information and coordination needs than older patients. Earlier studies have shown that patients with a higher education have different information-seeking patterns than those without a higher education. Patients with high education often find alternative information sources [19] and are more specific when requesting information [21].

We did not observe associations between gender and the experience of unmet needs. Other studies have demonstrated strong gender differences in the experience of needs, where women are more likely to express unmet needs [18,19,38]. Moreover, comorbidity has been shown to be associated with a high level of rehabilitation needs [20]. Surprisingly, in our study patients without comorbidity had a tendency to report unmet needs more frequently. A reason for this could be that patients with comorbidities may have had previous experiences with the health care system and might thus know how to handle needs. Furthermore, information on comorbidities in this study was obtained from patients’ self-report and could be underreported [39].

## Conclusion and implications

The findings in this study provide knowledge about cancer patients’ information and coordination needs in handovers, which is useful for improvements of the quality in cancer care [40]. The results suggest that approximately 82% of patients experience that information and coordination needs are met in their cancer care trajectory which is a relatively high level.

On the other hand, approximately every sixth patient reported unmet coordination needs and every sixth patient reported unmet information needs in handovers during the cancer care trajectory. Thus, there is still room for improvement especially regarding handovers between healthcare professionals and between healthcare professionals and patients.

## Competing interests

The authors declare that they have no competing interests. The authors alone are responsible for the content and the writing of the paper.
